# A Mobile Medication Support App and Its Impact on People Living With HIV: 12-Week User Experience and Medication Compliance Pilot Study

**DOI:** 10.2196/43527

**Published:** 2023-06-22

**Authors:** Mai Suzuki, Kou Yamanaka, Shinichi Fukushima, Mayu Ogawa, Yuki Nagaiwa, Toshio Naito

**Affiliations:** 1 Department of General Medicine Juntendo University Faculty of Medicine Tokyo Japan; 2 Shinjuku Higashiguchi Clinic Tokyo Japan

**Keywords:** human immunodeficiency virus, HIV, acquired immunodeficiency syndrome, mobile health, mHealth, medication compliance, satisfaction survey

## Abstract

**Background:**

The continuity of care between hospital visits conducted through mobile apps creates new opportunities for people living with HIV in situations where face-to-face interventions are difficult.

**Objective:**

This study investigated the user experience of a mobile medication support app and its impact on improving antiretroviral therapy compliance and facilitating teleconsultations between people living with HIV and medical staff.

**Methods:**

Two clinics in Japan were invited to participate in a 12-week trial of the medication support app between July 27, 2018, and March 31, 2021. Medication compliance was assessed based on responses to scheduled medication reminders; users, including people living with HIV and medical staff, were asked to complete an in-app satisfaction survey to rate their level of satisfaction with the app and its specific features on a 5-point Likert scale.

**Results:**

A total of 10 people living with HIV and 11 medical staff were included in this study. During the trial, the medication compliance rate was 90%, and the mean response rates to symptom and medication alerts were 73% and 76%, respectively. Overall, people living with HIV and medical staff were satisfied with the medication support app (agreement rate: mean 81% and 65%, respectively). Over 80% of medical staff and people living with HIV were satisfied with the ability to record medications taken (9/11 and 8/10 medical staff and people living with HIV, respectively), record symptoms of concern (10/11 and 8/10),and inquire about drug combinations (8/10, 10/10). And further, 90% of people living with HIV were satisfied with the function for communication with medical staff (9/10).

**Conclusions:**

Our preliminary results demonstrate the feasibility of the medication support app in improving medication compliance and enhancing communication between people living with HIV and medical staff.

## Introduction

According to recent statistics, there were an estimated 30,000 people living with HIV infections within Japan in 2018, and the diagnostic rate, antiretroviral therapy (ART) coverage rate, and viral suppression rate were around 86%, 80%, and 75%, respectively [1]. Engagement with HIV care may be hindered by stigma, confidentiality concerns, and insufficient support networks [2]. New measures are necessary to ensure continuity of care and to facilitate symptom and medication monitoring for people living with HIV between hospital visits, particularly during periods when access to HIV care services may be disrupted, such as during the COVID-19 pandemic [3]. Mobile-based apps have been shown to promote the link between HIV care at home and ART adherence [3-5], but reports of physicians’ experiences and testimonies regarding patient-centric apps are scarce [6]. This study investigated the user experience of a mobile medication support app that aims to improve ART compliance and facilitate teleconsultations between people living with HIV and medical staff. Preliminary user satisfaction results and the features resulting in higher satisfaction, as rated by medical staff and people living with HIV, were reported.

## Methods

### Participants

Two clinics (Shinjuku Higashiguchi Clinic and Juntendo University Hospital) were invited to participate in a 12-week trial of a mobile medication support app between July 27, 2018, and March 31, 2021. Eligible people living with HIV had to fulfill all of the following criteria: be diagnosed with HIV and be receiving ART; be aged between 20 and 90 years; have access to devices that can connect to the medication support app; be able to use the app unassisted; and provide written, informed consent. At project initiation, the medical staff registered eligible people living with HIV to use the app.

### Ethics Approval

All of the data used for this study were deidentified. Informed consent was obtained from all participants, and this study was approved by the Research Ethics Committee of Juntendo University Hospital (institutional review board number: 18-061).

### Medication Support App

The mobile medication support app, Medical Care Station (Embrace Co, Ltd), is a private social networking system that was developed for medical and nursing care use only. It was initially designed as a tool for communication between patients, patients’ families, and health care professionals in the home and medical care settings. The technical features of the app include direct patient–medical staff communication via text messages, daily medication reminders, and educational HIV information provision.

**Figure 1:**
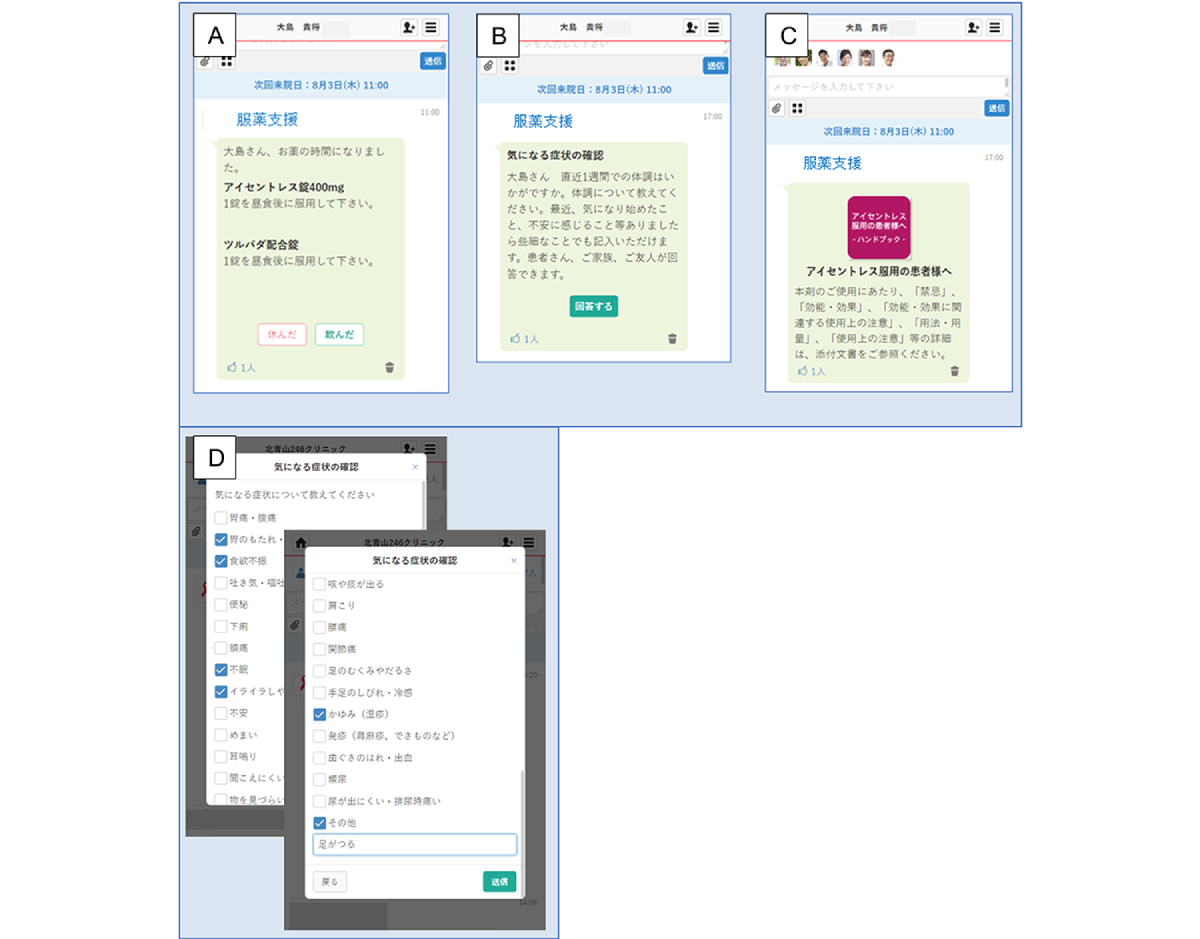
Representative screenshots of the user interface. (A) The message showing the time of medication and details of the medicine (the name of the medicine, dose, and timing). If the patient takes the pills, they click “done”; if no pills were taken, they click “skipped.” (B) The message asking about the patient’s condition during the week. The patient can send a message about their condition to health care professionals directly through this app. (C) By clicking a red icon, patients can access the information and education tool for antiretroviral therapy. (D) Representative screenshots of users recording symptoms of concern. When users have concerning symptoms, they can consult a physician directly by using this app. Users can either select from the list of symptoms or input the symptoms directly into the blank space, with descriptive text.

The five key functions of the app that were assessed in this study were (1) recording symptoms of concern, (2) recording medication status, (3) confirming drug combinations, (4) checking disease progress, and (5) providing teaching materials about the disease. Direct patient–medical staff communication included reporting symptoms of concern, inquiring about and confirming drug combinations, and checking the progression of disease conditions. The medication reminders were scheduled as each patient logged on to the app, and the time when patients took their pills was registered. A pop-up reminder appeared with an alert when it was time to take a medication; the medications taken were also recorded. The HIV education materials covered several topics, including the effectiveness and adverse events of each ART medication, the importance of good medical adherence, and nutritional information. Representative screenshots of the user interface within the app are shown in Figure 1.

### Outcome Measures

Data on the frequency and content of communications (ie, direct messages), baseline and posttreatment viral load, CD4 cell count, and ART compliance were collected. At the end of the 12-week period, users (people living with HIV and medical staff) were asked to complete the self-administered, in-app satisfaction survey to rate their level of satisfaction on a 5-point Likert scale (strongly agree, agree, neutral, disagree, and strongly disagree). The questionnaires included 1 section on the overall experience with the app and 5 subsections on the five key features of the app—(1) recording symptoms of concern, (2) recording medication status, (3) confirming drug combinations, (4) checking disease progress, and (5) distributing teaching materials about the disease. All data were collected and organized by using Microsoft Excel (Microsoft Corporation).

## Results

A total of 10 people living with HIV and 11 medical staff participated in this study. The mean age of people living with HIV was 44 years, and the average disease duration was 6.2 years (Table 1). A total of 12 educational notices requiring users to respond by selecting functions that appear on the screen or by texting were sent during the study period. The mean response rates to the notices for symptoms of concern and drug-drug interaction concerns were 73% and 76%, respectively. Specific symptoms of concern that were reported by people living with HIV in response to the notices are detailed in Table S1 in Multimedia Appendix 1. Concerns about drug-drug interaction were reported by 5 users, mainly on health supplements or remedies for sleep problems, colds, and depression (Table S2 in Multimedia Appendix 1). Overall, the mean total number of posts made by people living with HIV was 121.6 (range 19-143), averaging 10.1 posts per week (Table 1).

The average response time to daily medication reminders was approximately 4 hours, and the medication compliance rate was 90%, based on users’ (people living with HIV) responses to the ART reminders (Table 1). The mean CD4 cell counts at baseline and at 12 weeks were 491.9 (SD 190.5) cells per μL and 585.4 (SD 269.4) cells per μL, and after 12 weeks, viral loads decreased (2/10, 20%) or were undetected (7/10, 70%) in the majority of people living with HIV (Table 1). The CD4 cell count and viral load of each user before and after using the app are summarized in Table S3 in Multimedia Appendix 1.

**Figure 2:**
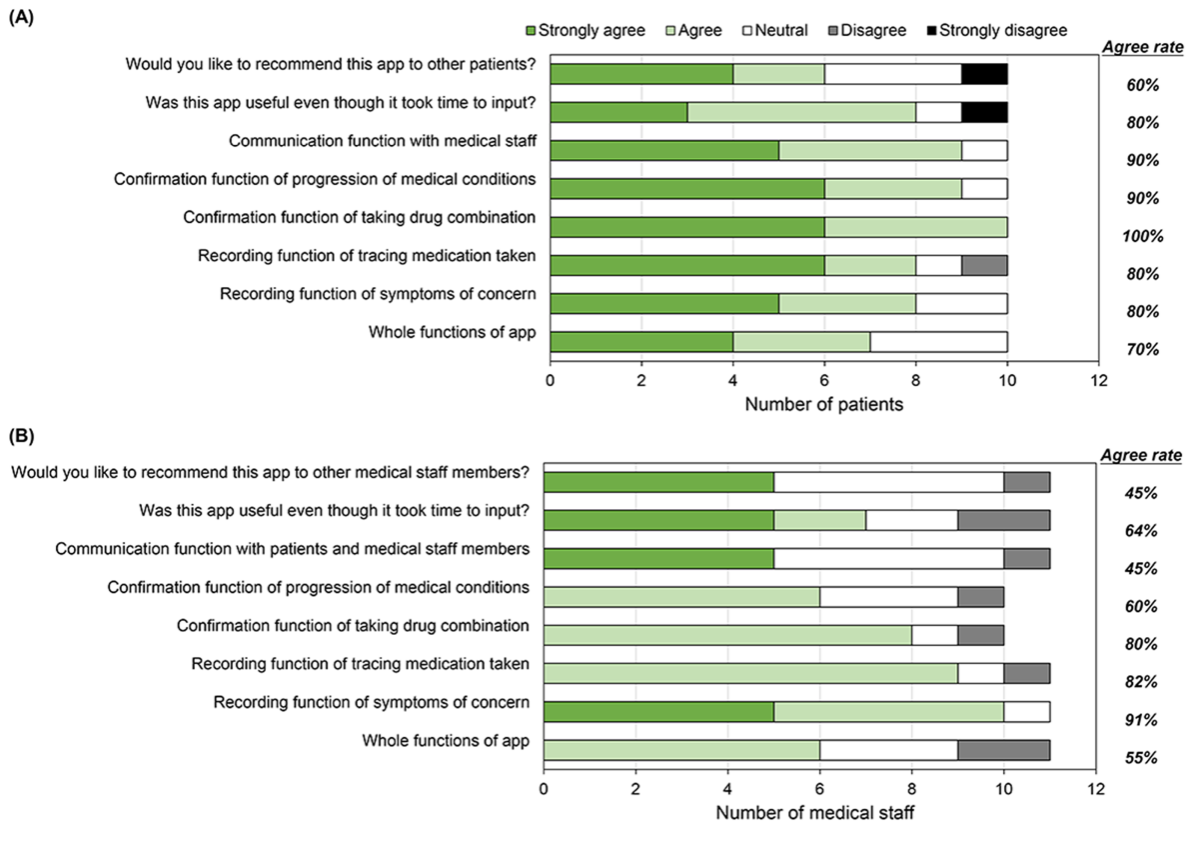
Agreement rate for each of the key features of the app. (A) Features most preferred by people living with HIV; (B) Features most preferred by medical staff.

Overall, people living with HIV and medical staff agreed that their overall experience with the app was satisfactory (agreement rate: mean 81% and 65%, respectively; Tables S4 and S5 in Multimedia Appendix 1). The agreement rate for each of the key features of the app is presented in Figure 2. The features most preferred by people living with HIV were the confirmation function for taking drug combinations (agree: 100%, 10/10), the confirmation function for the progression of medical conditions (90%, 9/10), and the function for communication with medical staff (90%, 9/10; Figure 2A). The features most preferred by medical staff were the recording function for symptoms of concern (91%, 10/11), followed by the recording function for tracking medications taken (82%, 9/11) and the confirmation function for taking drug combinations (80%, 8/10; Figure 2B).

**Table 1:** Patients’ (people living with HIV: N=10) characteristics and app usage–related outcomes.

Characteristics				Value
**Demographics**				
	Age (years), mean (SD; range)		44 (11.2; 27-65)	
	**Sex, n**			
		Male	9	
		Female	1	
	HIV duration (years), mean (SD; range)		6.2 (3.1; 0.3-9)	
**App usage (12 weeks), mean (range)**				
	Duration of using the app (days)		84.4 (84-85)	
	Response time to medication reminder (h:min)		3:57 (0:11-30:28)	
	Drug compliance^a^ (%)		90 (1.2-100)	
	Total number of posts		121.6 (19-143)	
	Number of posts made via the app per week		10.1 (1.6-­16)	
**Outcome measures**				
	**CD4 cell** **count (cells/μL), mean (range)**			
		Baseline	491.9 (201-821)	
		12 weeks	585.4 (170-957)	
	**Change in viral load among** **people living with HIV, n**			
		Increased	1	
		Decreased	2	
		Undetected	7	

^a^Based on responses to the reminder.

## Discussion

### Principal Findings

The HIV mobile health (mHealth) medication support app demonstrated promising results, with a large proportion of participating users—people living with HIV and medical staff—finding the key features to be satisfactory and worth recommending. The app’s abilities to provide alerts and track medications taken were found useful by both the people living with HIV and the medical staff in this study, since these not only allow people living with HIV to inquire about the safety or appropriateness of concomitant drug use but also assist medical staff in capturing data on the medication status and treatment adherence of people living with HIV. The medication compliance rate was 90%, based on users’ (people living with HIV) responses to the ART reminders in this study. Findings from randomized controlled trials and meta-analyses have shown that people living with HIV who receive weekly message reminder messages have significantly improved antiretroviral adherence and rates of viral suppression when compared with control individuals [4,5,7,8]. The app’s feature for facilitating direct patient-staff communication was welcomed by people living with HIV, and the underlying reason may be that the people living with HIV felt more comfortable and secure when a personal consultation with the medical staff was available. This was evident from the high agreement rates for the “I felt a sense of security to be monitored by medical staff” and “I felt a sense of security when I was able to consult at home” items for each of the app’s functions. Our study demonstrated the feasibility of this app in improving medication compliance and communication between people living with HIV and medical professionals.

The app’s function for recording the symptoms of concern was originally set up to assist medical staff in monitoring disease progression or improvement; nevertheless, it was also welcomed by people living with HIV. It is possible that this function assists people living with HIV to become more aware of their health status and, accordingly, explains the high agreement rate for the confirmation function for the progression of medical conditions*.*

The limitations of this pilot study include its small sample size and possible recruitment bias. Users who were comfortable with using mobile apps were more inclined to participate; therefore, the satisfaction rates among this study’s users may be overstated due to the users’ greater mobile app literacy. Although preliminary, it is encouraging to note that active users (people living with HIV) of the HIV app had positive improvements in both viral load and CD4 cell count. These findings are consistent with those of recent reports that also found that mHealth apps are feasible and acceptable interventions for improving ART adherence in people living with HIV and providing linkage to HIV care [9]. However, these findings should be confirmed in larger, well-controlled studies.

### Conclusion

Our medication support app may improve patient–medical staff communication and ART treatment compliance by providing the support needed for self-management and the remote monitoring of medical concerns among people living with HIV.

## Appendix

Multimedia Appendix 1 Supplement tables.

## Abbreviations

ART: antiretroviral therapy
mHealth: mobile health

## Data Availability

The data sets generated and analyzed during this study are not publicly available due to privacy and ethical restrictions. The data that support the findings of this study are included in this published article and its supplementary information files, where possible.
