# Supplementary material for: A Mobile Medication Support App and Its Impact on People Living With HIV: 12-Week User Experience and Medication Compliance Pilot Study
Source: JMIR Form Res. 2023 Jun 22;7:e43527. doi: 10.2196/43527 (PMC10337445; doi:10.2196/43527)
Supplement: Multimedia Appendix 1 [file formative_v7i1e43527_app1.docx]

**Table S1. Summary of PLWH users’ responses to educational notices on symptoms of concern**

| **Patient** | **Notices** | **Response rate (%)** | **Response** | **Having symptom** | **Average response time (hh: mm)** | **Symptoms reported** |
| --- | --- | --- | --- | --- | --- | --- |
| **1** | 12 | 8.3 | 1 | 1 | 41:03 | Rash (urticaria, rash, etc.) |
| **2** | 12 | 83.3 | 10 | 3 | 42:054 | Cough and sputum |
| **3** | 12 | 100.0 | 12 | 0 | 13:056 | No symptom |
| **4** | 12 | 58.3 | 11 | 7 | 00:09 | Symptoms: Nausea/vomiting/insomnia/  Rash (urticaria, rash  Insomnia/dullness/malaise/other/nausea/vomiting/headache  Facial dermatitis and stomatitis  Seborrheic dermatitis  Others: “*The discomfort and headaches have subsided. There were a few incidences on workdays, but there was almost no problem. I quit smoking and gained weight.*” |
| **5** | 12 | 0.0 | 0 | － | No response | No response |
| **6** | 12 | 100 | 12 | 2 | 09:52 | Stomachache, Diarrhea, Fatigue, sometimes dizziness-like symptom |
| **7** | 12 | 100 | 12 | 0 | 09:27 | No symptom |
| **8** | 12 | 91 | 11 | 0 | 13:38 | No symptom |
| **9** | 12 | 100 | 12 | 0 | 05:59 | No symptom |
| **10** | 12 | 91 | 11 | 4 | 05:58 | Stiffness of the chest |
| **Average** | 12 | 73 | 9 | 1.9 | 15:32* |  |

* Excluding PLWH #5. Abbreviation: PLWH, people living with HIV.

**Table S2. Summary of PLWH users’ responses to educational notices on DDIs and concomitant drug use**

| **Patient.** | **Notices** | **Response rate (%)** | **Response** | **Drugs other than ART medications** | **Average response time (hh: mm)** | **Drugs or supplements** | |
| --- | --- | --- | --- | --- | --- | --- | --- |
| **1** | 12 | 16.7 | 2 | 2 | 42:40 | Sleeping pills/antidepressant Valtrex |  |
| **2** | 12 | 83.3 | 10 | 10 | 13:14 | Cold Remedy/Supplement |  |
| **3** | 12 | 100.0 | 12 | 0 | 13:56 | None |  |
| **4** | 12 | 100.0 | 12 | 11 | 00:09 | 11 selections |  |
| **5** | 12 | 0.0 | 0 | － | No response | No response |  |
| **6** | 12 | 75 | 9 | 8 | 20:46 | Medicine for cold/Supplement/other: Pain killer/Intestinal regulator/Sleeping pill |  |
| **7** | 12 | 100 | 12 | 0 | 10:12 | None |  |
| **8** | 12 | 83.3 | 10 | 2 | 19:05 | Medicine for cold/Supplement |  |
| **9** | 12 | 100 | 12 | 0 | 10:35 | None |  |
| **10** | 12 | 100 | 12 | 0 | 06:05 | None |  |
| **Average** | 12 | 76 | 9 | 3.7 | 15:25* |  |  |

* Excluding PLWH #5.

Abbreviation: DDI, drug-drug interactions; PLWH, people living with HIV.

**Table S3. Each PLWH user’s ART medication record, CD4 counts, and viral load during the app trial period**

|  |  | **Baseline** | |  | **Week 12** | |
| --- | --- | --- | --- | --- | --- | --- |
| **Patient.** | **Medication** | CD4 count/μL | Viral load copies/mL |  | CD4 count/μL | Viral load copies/mL,  (Change) |
| **1** | DTG, TAF/FTC | 821 | 45 |  | 957 | 64 (↑)^a^ |
| **2** | DTG, TAF/FTC | 533 | 59 |  | 583 | Undetected |
| **3** | DRV/r, ABC/3TC | 674 | Undetected |  | 831 | Undetected |
| **4** | DTG, TAF/FTC | 588 | 21,000 |  | 919 | 99 (↓)^b^ |
| **5** | DAG/ABC/3TC | 530 | 870,000 |  | 567 | 150 (↓)^b^ |
| **6** | DTG, TAF/FTC | 555 | Undetected |  | 697 | Undetected |
| **7*** | DAG/ABC/3TC | 333 | Undetected |  | 380 | Undetected |
| **8** | DAG/ABC/3TC | 258 | Undetected |  | 258 | Undetected |
| **9** | DAG/ABC/3TC | 201 | Undetected |  | 170 | Undetected |
| **10** | DAG/ABC/3TC | 426 | 45 |  | 492 | Undetected |

Abbreviation: 3TC, lamivudine; ABC, abacavir; ART, antiretroviral therapy; DRV/r, darunavir boosted with ritonavir; DTG, dolutegravir; FTC, emtricitabine; SD, standard deviation; TAF, tenofovir alafenamide; PLWH, people living with HIV.

^a^ Viral load was increased from 45 to 64 copies/mL.

^b^ 2 PLWH had a very low viral load (<100 copies/mL) at baseline and had an undetectable viral load at 12 weeks.

**Supplementary Table S4. Agree rate for each sub-domain and total satisfaction survey results of PLWH (N=10)**

| Questionnaire | Agree rate |
| --- | --- |
| I: How did you feel about the ability to record “symptoms of concern” | 73% |
| There was a sense of security being monitored for symptoms of concern (at home, to medical staff) | 100% |
| I was able to convey the symptoms accurately (at the time of examination, to the medical staff) | 80% |
| I was aware of the changes in the symptoms and was able to respond appropriately. | 50% |
| I was aware of the change in the symptoms according to the medical staff's notice, I was instructed to respond appropriately (Emergency visit, etc.) | 60% |
|  |  |
| II: How did you feel about the ability to record “medication usage”? | 78% |
| I felt a sense of security being monitored through the record of medication usage (at home, to medical staff) | 90% |
| I was able to report/record medication usage accurately (at the time of examination, to the medical staff) | 100% |
| When I noticed that I missed a medication dose or made a mistake I was able to take appropriate action. | 70% |
| I was able to be instructed to take appropriate action by the medical staff when I told I forgot to take supplements (Emergency visit, etc.) | 50% |
|  |  |
| III: How did you feel about the function that allows you to check “drug combination”? | 53% |
| I felt a sense of security being monitored through the record of medication usage (at home, to medical staff) | 90% |
| I was able to consult (or communicate) about taking medicines and supplements (at the examination time, to medical staff) | 50% |
| I was aware I should be careful about taking medicine by myself through getting advice from medical staff about taking medicines and supplements | 40% |
| I was able to be taught to take appropriate medicine when medical staff noticed I was not taking drug combinations well. | 30% |
|  |  |
| IV: How did you feel about the ability to check the progress of your medical condition? | 85% |
| I felt a sense of security when the progress of medical conditions was being monitored (at home, or by medical staff) | 100% |
| I was becoming able to objectively recognize the progress of the medical condition | 90% |
| The medical staff immediately understood the situation at the time of consultation. | 90% |
| It became an opportunity to understand the treatment instruction and the medication instruction. | 60% |
|  |  |
| V: How did you feel about the function of “communication with medical staff” in general? | 78% |
| I felt a sense of security to be monitored by medical staff | 90% |
| I felt a sense of security when I was able to consult at home | 80% |
| I was able to consult the contents that are difficult to consult during a medical examination | 70% |
| I felt I received good advice after the consultation | 70% |
|  |  |
| Total: How did you feel about the entire app? | 81% |
| Whole functions of the app | 70% |
| Recording function of symptoms of concern | 80% |
| Recording function of tracing medication taken | 80% |
| Confirmation function of taking the drug combination | 100% |
| Confirmation function of progression of medical conditions | 90% |
| Communication function with medical staff | 90% |
| Was this app useful even though it took time to input? | 80% |
| Would you like to recommend this app to other patients? | 60% |

Agree rate: rate of patients’ response of strongly agree and agree.

Abbreviation: PLWH, people living with HIV.

**Supplementary Table S5: Agree rate for each sub-domain and total satisfaction survey results of medical staff (N=11)**

| Questionnaire | Agree rate |
| --- | --- |
| I: How did you feel about the ability to record “symptoms of concern”? | 61% |
| There was a sense of security that the state of symptom onset could be shared among medical staff (at home). | 73% |
| I was able to share symptoms accurately among medical staff (at the time of examination, to medical staff) | 73% |
| I could share with medical staff the changes in the symptoms reported by patients themselves and was able to respond appropriately. | 55% |
| The medical staff noticed changes in symptoms and were able to advise appropriately (emergency visit, etc.) | 45% |
|  |  |
| II: How did you feel about the ability to record “medication usage”? | 52% |
| I felt a sense of security that I could check the medication status at any time (at home). | 100% |
| I could share the medication status accurately among medical staff (at the time of medical examination, to medical staff) | 82% |
| I was able to share it among medical staff and respond appropriately about what patients noticed by themselves for forgetting to take medication or other mistakes | 18% |
| I was able to respond to take appropriate action when the medical staff noticed patients missed or used the wrong medications | 9% |
|  |  |
| III: How did you feel about the function that allows you to check “drug combination”? | 82% |
| There was a sense of security that the medication status could be shared among medical staff (at home). | 91% |
| I was able to share the medicines and supplements when talking with the medical staff | 91% |
| I was able to confirm and share the drug combinations with the medical staff | 82% |
| I was able to advise patients appropriately to take medicine when they were noticed to not be taking supplements well by medical staff | 64% |
|  |  |
| IV: How did you feel about the ability to check the progress of the medical condition? | 36% |
| There was a sense of security that I could check the progress of medical conditions | 64% |
| I was able to share progress among medical staff | 64% |
| Immediately share the situation between medical staff at the time of consultation | 9% |
| It was an opportunity to understand the treatment and medication instructions. | 9% |
|  |  |
| V: How did you feel about the function of “communication with medical staff” in general? | 39% |
| I felt a sense of security that the patient was being monitored by the medical staff | 55% |
| I was able to consult with patients even at home and share them among medical staff | 45% |
| I felt free to consult about contents that are difficult to consult during the medical examination. | 45% |
| I could share the consultation with medical staff and advise them well | 9% |
|  |  |
| Total: How did you feel about the entire app? | 65% |
| Whole functions of the app | 55% |
| Recording function of symptoms of concern | 91% |
| Recording function of tracing medication taken | 82% |
| Confirmation function of taking the drug combination | 80% |
| Confirmation function of progression of medical conditions | 60% |
| Communication function with patients and medical staff members | 45% |
| Was this app useful even though it took time to input? | 64% |
| Would you like to recommend this app to other medical staff? | 45% |

Agree rate: rate of medical staff’s response of strongly agree and agree.
